# Tuberculosis in Tigress

**Published:** 1891-05

**Authors:** 


### TUBERCULOSIS IN TIGRESS.

Cleopatra, a Bengal tigress 12 years old, weighing about 350 lbs. had been in confinement at the Menagerie, Central Park, New York, for 6 years and in perfect health until April 1st when she showed symptoms of illness, by refusing her food and general lasitude.

April 13th, she had become greatly emaciated, her skin was rough, the hairs had lost their lustre and stood on end; her nose was hot and dry; her face had a pinched, pained expression. She either lay in an awkward position in evident pain, or walked with her belly drawn up, and made attempts to stretch out, which she could not do. Her respirations, 30 to the minute, were labored, considerable movement of the thorax, being augmented by marked abdominal breathing. Auscultation near her mouth showed a tubular murmur, saliva flowed freely from her mouth and a muco-purulent discharge dropped from her nostrils. Her appetite was almost nil.

A diagnosis of double pneumonia was made, and pleuresy was excluded. As the lungs could not be auscultated, it remained uncertain if it was an idiopathic lobar pneumonia from cold, or a symptomatic lobular pneumonia due to tuberculosis or parasites. The cachectic condition of the animal rather pointed to tuberculosis and the prognosis was undoubtedly unfavorable.

As she would still take small pieces of meat, she was ordered small doses of iodide of potash to be given in them, with a pint of cod-liver oil and two drachms of tincture of iodine to be rubbed on the fore legs, which she licked off in her attempts to clean herself. Beef-tea in milk was also given.

On April 17th she appeared better, she had a less gaunt appearance, her hair had more lustre, she seemed more comfortable and her respirations were easier. She had eaten 14 lbs.of meat in the day and drank.

During the next few days she declined rapidly and died on April 22. Autopsy at College of Physicians and Surgeons same day by Dr. Geo. S. Huntington.

### AUTOPSY.

Trunk of *Filis tigris* received April 22nd. Head and extremities removed at Museum of Natural History.

BODY well nourished. *Voluntary Muscles* well developed and healthy.

THORAX.—*Heart* normal; post mortem clots in vena cava. *Lungs*: Lobar pneumonia, involving both lungs in all lobes. Complete hepatization. Scattered miliary tubucles.

ABDOMEN.— *Alimentary Canal*: Normal — nearly empty. *Liver*: A few scattered miliary tubucles. Slight enlargement of mesenteric glands. *Spleen* normal : *Pancreas* normal : *Right Ovary* one cist—size of walnut : *Left Ovary* normal. Corpora lutea in both : *Bladder* normal : *Kidneys* healthy.

